# Pediatric cochlear implantation: an update

**DOI:** 10.1186/s13052-014-0072-8

**Published:** 2014-09-02

**Authors:** Vincenzo Vincenti, Andrea Bacciu, Maurizio Guida, Francesca Marra, Barbara Bertoldi, Salvatore Bacciu, Enrico Pasanisi

**Affiliations:** Department of Clinical and Experimental Medicine, Unit of Audiology and Pediatric Otorhinolaryngology, University of Parma, Via Gramsci, 14 43126, Parma, Italy

**Keywords:** Sensorineural hearing loss, Cochlear implantation, Hearing rehabilitation, Surgery

## Abstract

Deafness in pediatric age can adversely impact language acquisition as well as educational and social-emotional development. Once diagnosed, hearing loss should be rehabilitated early; the goal is to provide the child with maximum access to the acoustic features of speech within a listening range that is safe and comfortable. In presence of severe to profound deafness, benefit from auditory amplification cannot be enough to allow a proper language development. Cochlear implants are partially implantable electronic devices designed to provide profoundly deafened patients with hearing sensitivity within the speech range. Since their introduction more than 30 years ago, cochlear implants have improved their performance to the extent that are now considered to be standard of care in the treatment of children with severe to profound deafness. Over the years patient candidacy has been expanded and the criteria for implantation continue to evolve within the paediatric population. The minimum age for implantation has progressively reduced; it has been recognized that implantation at a very early age (12–18 months) provides children with the best outcomes, taking advantage of sensitive periods of auditory development. Bilateral implantation offers a better sound localization, as well as a superior ability to understand speech in noisy environments than unilateral cochlear implant. Deafened children with special clinical situations, including inner ear malformation, cochlear nerve deficiency, cochlear ossification, and additional disabilities can be successfully treated, even thogh they require an individualized candidacy evaluation and a complex post-implantation rehabilitation. Benefits from cochlear implantation include not only better abilities to hear and to develop speech and language skills, but also improved academic attainment, improved quality of life, and better employment status. Cochlear implants permit deaf people to hear, but they have a long way to go before their performance being comparable to that of the intact human ear; researchers are looking for more sophisticated speech processing strategies as well as a more efficient coupling between the electrodes and the cochlear nerve with the goal of dramatically improving the quality of sound of the next generation of implants.

## Introduction

Hearing loss (HL) during the first 3 years of life can hinder speech and language acquisition with significant negative consequences on a child’s educational and psychosocial development. HL can be classified as conductive or sensorineural in nature; conductive hearing loss (CHL) is related to a disorder of the external and/or middle ear, which impacts on sound transmission toward the inner ear. Depending on the etiology of the CHL, the rehabilitation includes drug therapy, external and/or middle ear surgery, and hearing aids. Instead, sensorineural hearing loss (SNHL) affects the cochlea, which transforms sound vibration into a neural signal, or, the cochlear nerve, which transmits this signal to the auditory brain. Most SNHL is sensory and limited to the cochlea rather than neural [1]. The amount of SNHL is measured in decibels hearing level (dBHL) and classified in terms of pure tone average (PTA), the average of hearing thresholds for pure tone sounds, measured at 500, 1000, and 2000 Hz. Based on this classification, SNHL is ranked as mild (PTA between 21 and 40 dBHL), moderate (PTA between 41 and 70 dBHL), severe (PTA between 71 and 90 dBHL), and profound (PTA >90 dBHL). The treatment of SNHL varies depending on its severity and whether it affects one ear or both. Mild to severe SNHL can be successfully rehabilitated by means of hearing aids; however, in presence of profound SNHL, hearing aids, which merely make sound louder, may be not enough to properly understand and develop speech. In fact, SNHL cause sounds to become distorted and amplification through hearing aids makes them louder but not necessarily clearer. Under these circumstances, the best way for hearing and learning proper speech is represented by a cochlear implant (CI); this is an electronic device that bypasses the cochlea by means of an electrode array stimulating directly the cochlear nerve, thereby transmitting an electrical signal to the auditory cortex. Since their introduction more than 30 years ago, CIs have improved their performance to the extent that are now considered to be standard of care in the treatment of children with severe to profound SNHL loss [2]. Because of the incessant progress in the field of CIs, criteria for implantation continue to evolve within the pediatric population; at the same time, patient candidacy has broadened and changed over the years. The aim of this review was to take stock of the current indications, applications and outcomes in pediatric cochlear implantation.

## Background

SNHL is the most common congenital sensory deficit, with an incidence of one to three per 1000 live births; this incidence mounts up to 4-5% in neonates with risk factors for SNHL [3]. Genetic causes account for approximately 50% to 60% of pediatric SNHL, while 15% to 40% is due to an acquired cause, such as infections, ototoxic drugs, anoxia, low birth weight, hyperbilirubinemia, traumas, metabolic and autoimmune diseases [4–6]. A specific cause is still not identifiable in 15% to 30% of childhood SNHL [7].

In normal-hearing subjects, the sensory hair cells within the cochlea transform sound vibration into a neural signal; the latter is then transmitted via the cochlear nerve to the auditory cortex. CIs work by substituting these cells with electrodes that stimulate electrically the auditory nerve fibers. A CI has an external component, worn behind the ear just as a hearing aid, and an internal component, surgically embedded in the mastoid.

**Figure 1:**
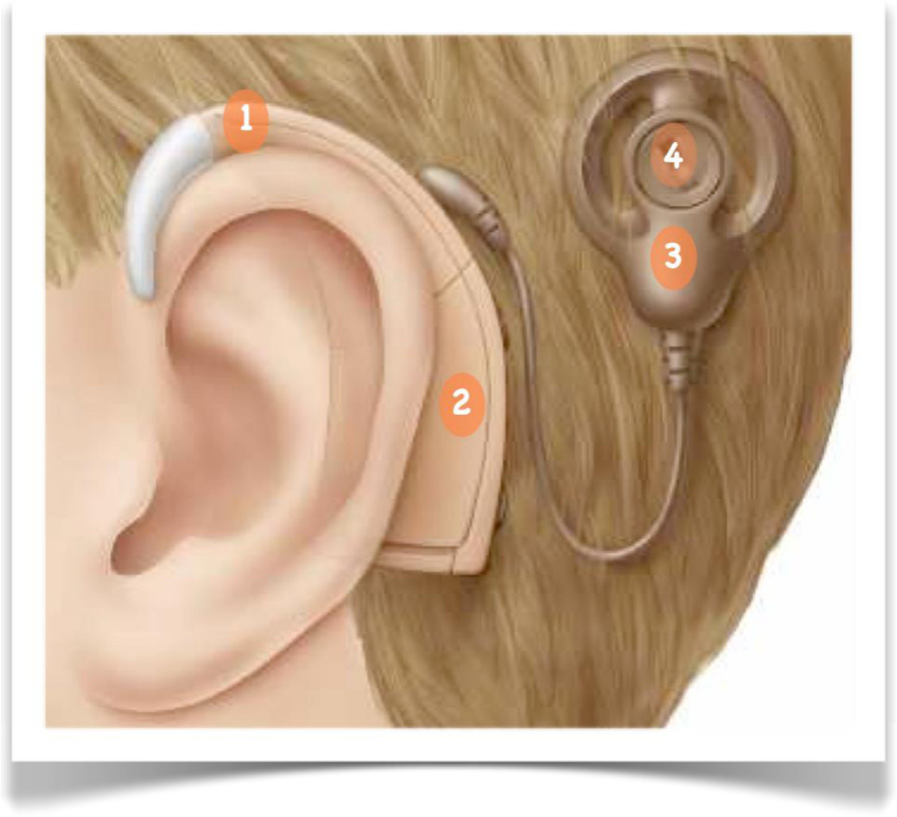
**Cochlear implant (external part).** 1: microphone; 2: speech processor; 3: external antenna; 4: magnet.

The external part (Figure 1) consists of a microphone for obtaining sound, a speech processor that analyzes and encodes sound into a digital code, and a magnetic headpiece transmitting the coded signal to the internal part via a transcutaneous radiofrequency link to the internal part.

**Figure 2:**
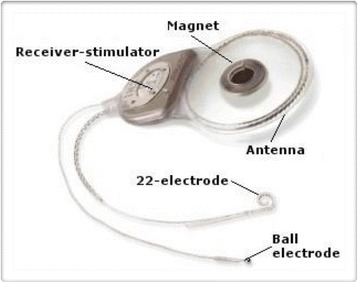
**Cochlear implant (internal part).**

**Figure 3:**
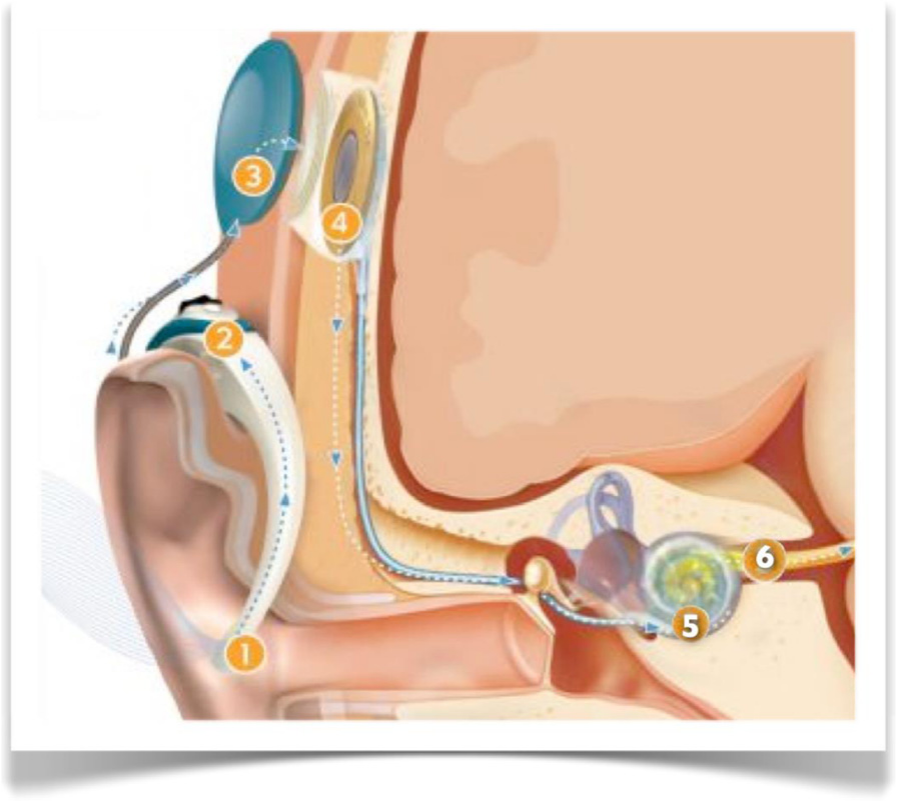
**How a cochlear implant works.** The microphone (1) picks up sound; the speech processor (2) analyzes and transforms sound into digital information; the magnetic headpiece (3) transmits the coded signal to the surgically implanted part (4); the intracochlear electrodes (5) stimulate the cochlear nerve fibers (6).

The internal part (Figure 2) contains a receiver-stimulator (having a size as a pacemaker) that receives and decodes the data, and in turn sends the decoded signal to the electrode array. The latter is the core of the system and consists of a flexible silicone carrier containing a variable number of electrodes. The electrode array is surgically inserted into the scala tympani of the cochlea and stimulates directly the residual cochlear nerve fibers. On the European market, there are four types of CIs that differ in design, shape and number of electrodes, strategy of speech processing, accessories. Figure 3 shows how a cochlear implant works.

### Candidacy evaluation, patient selection and surgical notes

Several factors must be considered to establish whether a child is suitable for implantation; thus, the patient selection is of outstanding importance for a successful cochlear implantation. A complete evaluation should comprise a series of tests, including audiologic, medical and imaging studies, as well as speech and language evaluation; furthermore, patient/family counseling is fundamental to explain them the potential benefits and to create realistic expectations.

First of all, the existence of the current audiologic criteria for candidacy to cochlear implantation needs to be verified. Pediatric audiologic evaluation is based on several subjective and objective hearing tests. Taken together, these tests aim at accurately identifying the degree of HL across the audiometric frequencies. The current guidelines indicate that patients until the age of 2 years should have a bilateral profound SNHL (PTA for 500, 1000 and 2000 Hz >90 dBHL), while children older than 2 years should have a severe to profound SNHL (PTA for 500, 1000 and 2000 Hz >75 dBHL) [8,9]. Furthermore, cochlear implantation should be performed after verifying insufficient benefits from appropriate binaural hearing aids and intensive aural rehabilitation. The preoperative speech and language evaluation is equally important for the decision-making process, as well as to monitor the appropriateness of the rehabilitation program; auditory performance, speech production and mode of communication should be assessed by means of different tests appropriately selected according to hearing impairment, chronological age and cognitive status. In terms of onset, a hearing loss may be defined as prelingual (the hearing impairment occurred before the acquisition of spoken language skills, that is before the age of 2 years), postlingual (the hearing impairment occurred after the acquisition of speech and language, that is after the age of 5 years), or perilingual (the hearing impairment occurred after acquiring some spoken language but before acquisition was complete, that is between 2 and 5 years).

The medical evaluation aims at verifying whether the child is healthy enough to withstand general anesthesia and surgical procedure. Furthermore, since the risk of developing meningitis is higher in children with SNHL (implanted or not) than those one without SNHL, immunization history should be confirmed; age-appropriate immunization should be updated at least 2 weeks before implantation according to published recommendations [8].

Preoperative imaging assessment is mandatory to verify the presence of the minimal requirements for cochlear implantation, i.e. a patent cochlea and an intact cochlear nerve. Imaging is crucial to identify findings that may complicate or require modifications of the surgical technique, such as cochleo-vestibular malformations, middle ear and facial nerve anomalies, and cochlear obstruction. Finally, the radiological study can contribute in choosing which ear may be more suitable for implantation. High resolution computed tomography (HRCT) provides information regarding the structure of the bony labyrinth, the number and the patency of the cochlear turns, the size of the internal auditory canal (IAC), the position of the facial nerve and the vascular structures, and the anatomy of the middle ear and mastoid. Magnetic resonance imaging (MRI) is useful in confirming the presence of the cochlear nerve, as well as in searching for central auditory pathway abnormalities and fibrous obliteration of the membranous labyrinth. Often, both imaging modalities are performed in pediatric candidates, but the protocol varies among the different cochlear implant centers [8]. A trade off between the need to cut costs and to avoid unnecessary radiations to children and a detailed radiological study, may be represented by performing MRI as initial modality, and reserving HRCT to children in which cochleovestibular anomalies, cochlear obstruction or narrowing of the IAC are identified. Current contraindications for cochlear implantation are summarized in Table 1.

**Table 1:** **Current contraindications for cochlear implantation**

**Absolute**	**Relative**
Absence of cochlear development	Aplasia of the acoustic nerve
Deafness due to lesions of the central auditory pathway	Medical conditions or developmental delays that would severely limit partecipation in aural habilitation
Massive cochlear ossification that prevents electrode insertion	

**Figure 4:**
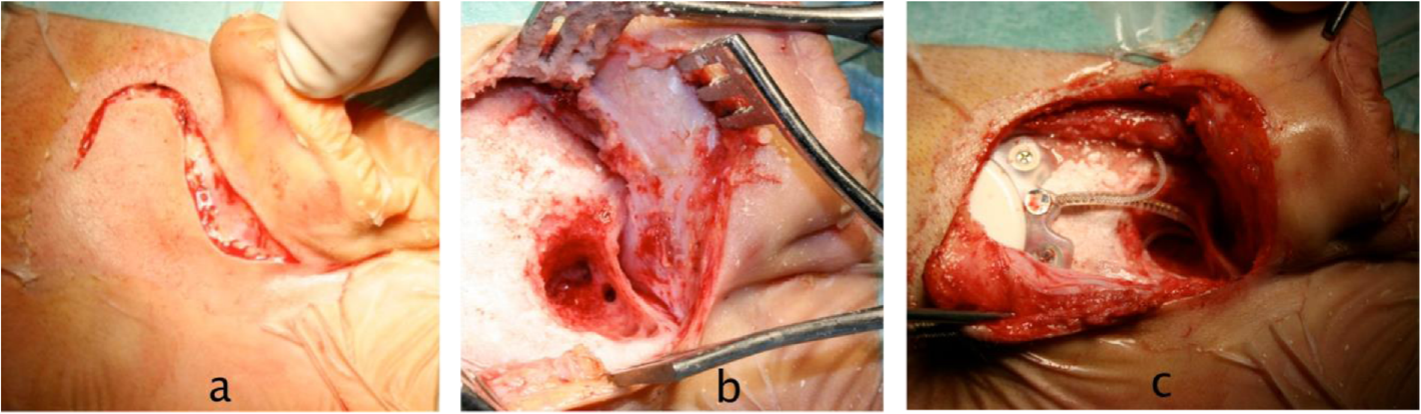
**Cochlear implant surgery. a**: skin incision; **b**. mastoidectomy; **c**: cochlear implant secured in place.

Surgery is done under general anesthesia and it is usually well tolerated. CIs are placed through small skin incision in the retroauricolar region (Figure 4a); a surgical opening (mastoidectomy) is made in the mastoid to provide access to the cochlea from behind (Figure 4b). Once identified the round window, the latter is opened and the electrode array is inserted into the cochlea. After the implant has been secured in place and before closing the surgical access (Figure 4c), intraoperative electrophysiological testing is performed to verify the correct functioning of the device and to record the neural responses to the electrical stimuli. In standard cases, the procedure takes about 2 hours; children are generally discharged from hospital within 2–3 days. Cochlear implantation has a low rate (about 10%) of complications; major complications are rare, accounting for only 20 to 30% of all complications on average [8], and include facial nerve injury (0.39%), perilymphatic gusher/cerebrospinal fluid fistula (0.25%), and meningitis (0.11%). The most frequent complications are temporary taste disturbance, wound infections, and device failure. The activation of the implant is usually done 2–4 weeks after surgery, when healing is complete, and consists in setting the sound levels presented to each electrode within the cochlea. During the first year after activation, the cochlear implant is periodically tuned according to the child responses in order to maintain optimal stimulation levels.

## Discussion

Modern multichannel CIs were introduced in the late 1970s; only post-lingually deafened adults were considered candidates for implantation. As consequence of the excellent results achieved in adults and after the safety of the device had been confirmed, in 1990 FDA approved the use of CIs in congenitally deafened children; since then, the candidacy criteria in pediatric age have changed and expanded over the years.

As for the ideal age for implantation in presence of congenital deafness, there has been a growing trend to decrease it. The lack of auditory information from the environment during early childhood impedes the normal development of the auditory system, and interferes with the acquisition of language skills. In fact, there is a window of time in the first 3 years of life (the “sensitive period”), during which the child’s brain is very plastic and has the ability to develop new neural pathways in response to auditory stimuli [10]. Behind this period, the auditory cortex can no longer be recruited by auditory input because the intact senses take over the auditory neural network through a process of cross-modal reorganization [11]. The rationale behind early cochlear implantation is to minimize the consequences related to sensory deprivation during the sensitive period. Several studies have shown that children implanted before 2 years of age perform significantly better than children implanted at older ages [8,12,13]. In presence of bilateral profound SNHL and limited benefit from appropriate binaural hearing aids, the goal is to implant, as soon as possible, bearing in mind that the inferior limit is also conditioned by surgical and anesthesiological risks [14]. Currently, the FDA has approved cochlear implantation in 12 months and older children, but some centers are implanting infants as young as 6 months [13]. Colletti et al. [15] reported on 12 children implanted at or before the age of 6 months; four years after implantation, these children had receptive and expressive language skills similar to normal-hearing peers. However, other studies did not confirm clear evidence of improved outcomes in children implanted in the first year of life compared with those implanted a year later [16]. In addition, a major concern in performing early implantation for very young infants is the reliability of the audiologic tests. Children with SNHL from meningitis deserves special attention because a postinfectious inflammatory process, the so-called labyrinthitis ossificans, can lead, in a few months, to a cochlear obstruction with fibrous or bony tissue that makes electrode insertion more challenging or even impossible [14]. For this reason, in these cases it is often necessary to expedite surgery.

Recently, cochlear implant candidacy has been extended also to some children with significant residual hearing [17]. An example is a bilateral and asymmetric SNHL, in which significant benefit can derive from cochlear implantation in the worse hearing ear in combination with a hearing aid in the better ear [18]. The combination of acoustic and electrical stimulation, in the same ear, can be applied in children with the so-called “ski-slope hearing loss”, where low frequencies up to 1000 Hz are well-preserved, while a severe to profound hearing loss is present in the frequencies over 1000 Hz [12]. Under these circumstances, hearing aids fail to provide adequate amplification of the high frequencies necessary for oral communication. Specially manufactured short electrodes or conventional electrodes inserted only in the high frequency area of the cochlea by means of “soft surgery” can allow preservation of residual hearing; this situation offers the possibility to transmit the high frequency information through an electrical stimulation, and the low frequency information through an acoustic stimulation [19].

Inner ear malformations (IEMs) (Table 2) are present in approximately 20% of children with congenital SNHL [20,21]. Initially, IEMs were considered as a contraindication to cochlear implantation, mainly because histopathologic reports showed decreased and irregularly distributed neural tissue in the malformed cochleas [22]. Moreover, in presence of temporal bone anomalies, the surgeon may be confronted with several challenging problems, including cerebrospinal fluid gusher, facial nerve injury, and electrode misplacement into the IAC [12]. The increased experience in the surgical techniques [23,24], as well as the progressive advancement of cochlear implant technology, has led to more children with IEMs to be considered as candidates. The review of the literature showed that functional outcomes are inversely correlated with the severity of malformation [12,23]. Mild anomalies, such as enlarged vestibular aqueduct or incomplete partition, are usually associated with outcomes similar to those of children without malformations. Severe abnormalities, such as common cavity, cochlear hypoplasia, stenotic IAC, and cochlear nerve deficiency, also benefit from implantation, but with poorer performance [25,26]. Other important prognostic factors include progressive SNHL, duration of implant use and the presence of additional handicaps [27].

**Table 2:** **Cochleo-vestibular malformations as classified by Sennaroglu and Saatci** [20]

**Malformation**	**Characteristics**
Labyrinthine aplasia	Complete absence of cochlea and vestibule
Cochlear aplasia	Cochlea absent, vestibule present
Common cavity	Cochlea and vestibule are represented by a single, undifferentiated cystic cavity
Incomplete partition type 1	Cystic-appearing cochlea lacking entire modiolus and cribriform area; large cystic vestibule
Cochlear hypoplasia	Cochlea and vestibule smaller than normal
Incomplete partition type 2	Cochlea consists of 1.5 turns (normally it has 2.5 turns); middle and apical turns coalesce to form a cystic apex

More than 30% of children with SNHL have additional disabilities, such as developmental delay, autism and attention deficit hyperactive disorders, cerebral palsy, visual impairment, mental retardation [28]. Although certain such patients develop speech perception and language skills, additional disabilities are usually associated with poor performance in terms of development of oral communication [29–31]. However, in this specific population of patients, parameters of success are hard to define. Families of children with disabilities often perceive significant benefits from cochlear implantation in terms of awareness of the environment, improved interactions with peers, vocalization and developing speaking skills [32,33]. Based on the possibility to improve their quality of life, actually many centers offer cochlear implantation to children with additional disabilities, once verified that parental expectations are appropriate and realistic. Anyway, these children require complex and individualized therapy to maximize benefit from cochlear implantation [28].

Binaural hearing is fundamental in the localization of the sound as well as in listening in noisy environments. Children with unilateral cochlear implant and little or no usable hearing in the non-implanted ear show deficiency in both tasks because of the lack of binaural hearing [12]. In order to improve performance, binaural cochlear implantation has been introduced over the past decade. Bilateral cochlear implantation may be performed during either a single (simultaneous) or two different (sequential) interventions. Both techniques have been shown to be effective; however, if a sequential strategy is chosen, the inter-stage interval has to be preferably comprised between 6 and 12 months to maximize the performance with the second implant [34]. It has been recognized that children with bilateral CIs have improved speech understanding in noise and better abilities to localize sound, both in quiet and in noise, than children with one implant only [2,35]. In addition, bilaterally implanted patients develop complex expressive and receptive spoken language, and use significantly more audition and vocalization in communication in the early postimplantation period than their unilaterally implanted peers [36–38]. Finally, bilateral cochlear implantation promotes the development of the central auditory system through bilateral cortical stimulation, ensures that the better ear is always implanted and provides recipients to have a backup if one device fails or malfunctions [39]. Although there are also some disadvantages in choosing bilateral implantation, including increased time of surgery or two separate surgeries, increased expenses, and inability to preserve one ear for newer therapies, numerous authors agree that the pros often outweigh the cons [14,35,36].

## Conclusions

Cochlear implantation is worldwide considered a safe and highly effective technique in rehabilitating children with severe to profound SNHL. To understand the improvements made within CIs, it suffices to note that twenty years ago cochlear implant recipients were usually compared with age-matched deaf children who used high-powered hearing aids as controls; nowadays, they are compared with age-matched normal-hearing children. Benefits include not only better abilities to hear and to develop speech and language skills, but also improved academic attainment, improved quality of life, and better employment status. As a consequence, the society also derives benefit from cochlear implantation; such rehabilitation technique is costs-saving by cutting educational costs and restoring the work productivity potential. Early intervention and a short duration of deafness before implantation are associated with the best language acquisition results, taking advantage of sensitive periods of auditory development. When clinically appropriate, bilateral cochlear implantation should be considered for the advantages it offers in terms of sound localization and speech understanding in noisy environments. Advancement of technology, improvement of surgical techniques, as well as increased understanding of auditory system, allowed candidacy criteria for pediatric cochlear implantation to be expanded; currently, more deaf children with various adverse conditions can benefit from this revolutionary technique. However, it is necessary to always bear in mind that CIs have their limitations since these devices allow deaf people to become integrated in the hearing world but do not restore normal hearing; in addition, outcomes still vary among patients. It is hoped that future researches will improve the delivering of the fine-structure content of the speech signal and responsiveness of the brain to electrical stimulation.
